# Record linkage research and informed consent: who consents?

**DOI:** 10.1186/1472-6963-7-18

**Published:** 2007-02-12

**Authors:** Nicole Huang, Shu-Fang Shih, Hsing-Yi Chang, Yiing-Jenq Chou

**Affiliations:** 1 Institute of Public Health, School of Medicine, National Yang-Ming University, No. 155, Section 2, Li-Nong Street, Taipei 112, Taiwan, R.O.C; 2 Center for Health Policy Research and Development, National Health Research Institutes, No. 35, Keyan Road, Zhunan Town, Miaoli County 350, Taiwan, R.O.C

## Abstract

**Background:**

Linking computerized health insurance records with routinely collected survey data is becoming increasingly popular in health services research. However, if consent is not universal, the requirement of written informed consent may introduce a number of research biases. The participants of a national health survey in Taiwan were asked to have their questionnaire results linked to their national health insurance records. This study compares those who consented with those who refused.

**Methods:**

A national representative sample (n = 14,611 adults) of the general adult population aged 20 years or older who participated in the Taiwan National Health Interview Survey (NHIS) and who provided complete survey information were used in this study. At the end of the survey, the respondents were asked if they would give permission to access their National Health Insurance records. Information given by the interviewees in the survey was used to analyze who was more likely to consent to linkage and who wasn't.

**Results:**

Of the 14,611 NHIS participants, 12,911 (88%) gave consent, and 1,700 (12%) denied consent. The elderly, the illiterate, those with a lower income, and the suburban area residents were significantly more likely to deny consent. The aborigines were significantly less likely to refuse. No discrepancy in gender and self-reported health was found between individuals who consented and those who refused.

**Conclusion:**

This study is the first population-based study in assessing the consent pattern in a general Asian population. Consistent with people in Western societies, in Taiwan, a typical Asian society, a high percentage of adults gave consent for their health insurance records and questionnaire results to be linked. Consenters differed significantly from non-consenters in important aspects such as age, ethnicity, and educational background. Consequently, having a high consent rate (88%) may not fully eliminate the possibility of selection bias. Researchers should take this source of bias into consideration in their study design and investigate any potential impact of this source of bias on their results.

## Background

Linking computerized health insurance records with routinely collected survey data is becoming increasingly popular in health services research. Record linkage serves as a powerful tool to augment and validate the data acquired for research. Inevitably, the growing number of record linkage studies have created a serious public concern regarding the privacy of personal information, and has led many governments to institute a mandatory informed consent for access to health records [1]. However, if consent is not universal, the requirement of written informed consent may introduce a number of research biases. Most importantly, seeking individual informed consent may lead to serious selection bias, and may compromise the external validity of the research if the consent pattern is not uniform. Furthermore, since not everyone interviewed in surveys gives consent to access their health records, the sample size needs to be increased to compensate for those that do not consent.

Only a few studies have examined patterns of consent in relation to linkage of survey data with medical or administrative records [2-8]. The findings of these studies indicate that consent is not universal, and that factors such as age [2-5,8], gender [2,3], health status [2-4,6-8], socioeconomic status [4-7], and ethnicity [5] may have influenced consent to record linkage. However, the direction of the relationship between these factors and the giving of consent remains inconclusive. For example, studies conducted in the USA [3,8], and Australia [4] suggest that older people are more likely to give consent, while studies in the UK [2,5] suggest the opposite, and still others [6] show no influence. These variations in data may be owing to differences in study design and the target population. Previous researches are, for the most part, limited to patients with a particular disease, specific demographic sub-populations (mostly the elderly or women), or to Western countries. No one has investigated this important issue for a general population in an Asian country, even though record linkage studies conducted in Asia play an increasingly important role in medical and health research. Due to cultural heterogeneity regarding confidentiality and privacy issues, the experiences in western countries may not be generalizable to Asian populations. This study aimed to explore how, in Taiwan, a newly developed Asian country with rich data resources, individuals in the general adult population who consent differ in important aspects from those who decline.

## Methods

In 2001, the National Health Research Institutes (NHRI) in Taiwan conducted the National Health Interview Survey (NHIS). The NHRI used a multistage stratified systematic sampling design based on the degree of urbanization, geographic location, and administrative boundaries to select a representative sample. Details of the design and sampling scheme have been reported elsewhere [9]. The consent rate of the original health survey was 94.2% and the demographic characteristics of this sample were consistent with the population [9]. In this survey, a national representative sample of 22,121 people aged 12 years and older was interviewed. For the purpose of this study, we only included those 15,413 survey respondents with a valid 10-digit personal identification (ID) number and who were aged 20 years or older. Like the social security number in the United States, each resident in Taiwan has a personal ID number that is provided by the government. During the survey process, some personal ID numbers might have been miscoded or missing. Only those ID numbers which were not missing and were consistent with the official algorithm of personal ID assignment were considered "valid" identification numbers.

At the end of the NHIS survey, the participants were asked for permission to access their National Health Insurance (NHI) records for research purposes. The NHI program is a mandatory national health insurance program which provides comprehensive medical care coverage to all civilian Taiwanese residents, and the enrollment rate is above 99%. This comprehensive benefits package includes preventive care, ambulatory care in both clinic and hospital settings, inpatient hospital services, dental services, Chinese medicine services, and prescription drugs. The NHI claims data contains a government assigned personal ID number, gender, date of birth, date of the service, diagnoses, procedures, drugs prescribed, reimbursements, hospital and physician identifiers. Those participants that consented to linkage were asked to sign a consent form. In addition to the question on consent, the answers, to a large range of other survey questions provided by these participants allowed us to identify the factors related to this consent. The survey variables considered in this study include demographics (age, gender, marital status, ethnicity, urbanization of residence), socio-economic status (education, household income), and health status (the 8 health domains from the 36-Item Short-Form Health Survey Taiwan version 1.0-physical functioning, role limitation-physical, role limitation-emotional, mental health, social functioning, bodily pain, vitality, general health). A higher domain score reflects better health or functioning.

Both simple and multiple logistic regression models were used. First, a series of simple logistic models were fitted separately for each factor. Then, a multiple logistic model was used to relate the adjusted odds of consent with all demographics, socioeconomic status, and health status variables. All analyses were conducted using SAS 9.0 and STATA 8.0 statistical software packages.

## Results and discussions

Of the 15,413 individuals with a valid ID, 802 interviewees with incomplete survey information were excluded. Of the 802 that were excluded, 74% had given consent. The remaining sample of 14,611 individuals was used for analyses. Of the 14,611 NHIS participants, 12,911 (88%) gave consent to link their questionnaire to their NHI records, and 1,700 (12%) denied consent (Table 1). A high percentage of participants gave consent to link their health insurance records to their questionnaire results. Non-consent was the highest among those aged 65 or older, who were married, illiterate, those with a monthly household income < 30,000 New Taiwan (NT) dollars, or were living in a suburban area. Non-consenters had relatively lower mean scores in all eight physical and mental functional status domains of SF-36. Based on the distributions presented in the Table 1, the consent rates appeared to differ for all characteristics, except for gender.

**Table 1:** Characteristics of study sample

**Characteristics**	Informed consent			
	
	Yes (n = 12911)		No (n = 1700)	
	
	No.	(%)	No.	(%)
**Age**				
20–24	1605	(90.7)	164	(9.3)
25–34	2974	(91.1)	289	(8.9)
35–44	3071	(89.4)	365	(10.6)
45–54	2443	(87.8)	339	(12.2)
55–64	1366	(85.1)	240	(14.9)
65–74	1010	(83.2)	204	(16.8)
>=75	442	(81.7)	99	(18.3)
**Gender**				
Male	6397	(88.9)	798	(11.1)
Female	6514	(87.8)	902	(12.2)
**Marital Status**				
Other	3065	(90.9)	307	(9.1)
Married	9846	(87.6)	1393	(12.4)
**Ethnicity**				
Fujianese	9870	(88.0)	1350	(12.0)
Hakka	1207	(89.5)	141	(10.5)
Indigenous people	347	(96.9)	11	(3.1)
Others	1487	(88.2)	198	(11.8)
**Education**				
College and above	3403	(90.1)	373	(9.9)
Senior high school	3920	(89.8)	446	(10.2)
Junior high school	2033	(89.7)	233	(10.3)
Elementary school	2560	(87.0)	382	(13.0)
Illiterate	995	(78.9)	266	(21.1)
**Household Income**				
≥NT$100,000	2309	(90.1)	255	(9.9)
NT$70,000-NT$99,999	2462	(89.1)	302	(10.9)
NT$50,000-NT$69,999	2795	(89.1)	341	(10.9)
NT$30,000-NT$49,999	2911	(88.6)	376	(11.4)
<NT$30,000	2434	(85.1)	426	(14.9)
**Urbanization**				
Urban area	7271	(89.6)	847	(10.4)
Suburban area	2398	(84.9)	426	(15.1)
Rural area	3242	(88.4)	427	(11.6)
**SF-36 Health Status Measures**	**Mean**	**(SD)**	**Mean**	**(SD)**
Physical functioning	91.6	(16.5)	88.4	(20.2)
Role-physical	83.0	(33.9)	78.1	(38.0)
Role-emotional	79.8	(36.0)	77.2	(38.0)
Social functioning	86.7	(17.2)	84.9	(18.6)
Bodily pain	81.6	(21.1)	79.6	(22.8)
Vitality	67.8	(18.6)	64.1	(20.0)
Mental health	73.1	(16.7)	71.4	(17.2)
General health	72.6	(19.1)	70.3	(19.7)

More specifically, Table 2 reports the main results of both the simple and the multiple logistic regression analyses. The bivariate results indicate that factors such as age, marital status, ethnicity, education, household income, urbanization and functional status were significant predictors of non-consent. But, after adjusting for other factors, the multiple logistic regression analysis showed a dose-response between non-consent and increased age, which is consistent with other studies [2,5,8]. On the other hand, after adjusting for age, gender, household income, and functional status, the minority ethnic group in Taiwan (Taiwan aborigines) was 77% less likely to refuse than the majority ethnic group (Fujianese). Although the observations for ethnic minorities as reported by other studies in the USA and the UK were either not different from the general population [3,6] or more likely [5] to refuse consent, we observed the opposite in Taiwan. This is possibly due to the heterogeneity in culture or a lower suspicion of health research. In terms of socioeconomic status (SES), we found that, consistent with other studies [4-7] those refusing consent were more likely to be illiterate (odds ratio = 1.52, 95% confidence interval = 1.19–1.94), and with monthly household income lower than $NT 30,000 (odds ratio = 1.24, 95% confidence interval = 1.04–1.49). Also, individuals living in suburban areas (odds ratio = 1.45, 95% confidence interval = 1.27–1.65) were significantly more likely to refuse consent compared to those living in urban areas. Most importantly, contrary to other studies in the USA [2,8], the UK [3], and Australia [4], in the present study physical and mental health did not have a strong influence on giving consent or on refusing consent among Taiwanese adults. Out of the eight SF-36 health domains, only the vitality domain showed a marginally significant influence on non-consent (odds ratio = 0.91; 95% confidence interval = 0.88–0.95).

**Table 2:** Factors associated with refusing consent to link questionnaire with health insurance records

**Characteristics**	Unadjusted OR (95% CI)		Adjusted OR (95% CI)	
**Age**				
20–24	1.00		1.00	
25–34	0.97	(0.80 – 1.17)	0.92	(0.74 – 1.15)
35–44	1.18	(0.98 – 1.42)	1.09	(0.86 – 1.39)
45–54	1.36	(1.12 – 1.64)	1.24	(0.95 – 1.61)
55–64	1.73	(1.41 – 2.12)	1.33	(1.00 – 1.79)
65–74	2.00	(1.63 – 2.47)	1.41	(1.03 – 1.92)
>=75	2.21	(1.74 – 2.82)	1.39	(0.96 – 2.01)
**Gender**				
Male	1.00		1.00	
Female	1.07	(0.97 – 1.18)	1.02	(0.92 – 1.14)
**Marital Status**				
Other	1.00		1.00	
Married	1.38	(1.22 – 1.56)	1.08	(0.90 – 1.29)
**Ethnicity**				
Fujianese	1.00		1.00	
Hakka	0.84	(0.70 – 1.00)	0.90	(0.75 – 1.08)
Indigenous people	0.23	(0.13 – 0.40)	0.23	(0.12 – 0.42)
Others	0.96	(0.83 – 1.12)	1.05	(0.89 – 1.25)
**Education**				
College and above	1.00		1.00	
Senior high school	1.06	(0.92 – 1.22)	0.98	(0.85 – 1.15)
Junior high school	1.09	(0.93 – 1.29)	0.91	(0.75 – 1.10)
Elementary school	1.39	(1.20 – 1.61)	1.01	(0.83 – 1.22)
Illiterate	2.36	(2.01 – 2.78)	1.52	(1.19 – 1.94)
**Household Income**				
≥NT$100,000	1.00		1.00	
NT$70,000-NT$99,999	1.10	(0.93 – 1.30)	1.09	(0.91 – 1.30)
NT$50,000-NT$69,999	1.11	(0.94 – 1.31)	1.06	(0.89 – 1.26)
NT$30,000-NT$49,999	1.17	(1.00 – 1.38)	1.11	(0.93 – 1.32)
<NT$30,000	1.60	(1.36 – 1.87)	1.24	(1.04 – 1.49)
**Urbanization**				
Urban area	1.00		1.00	
Suburban area	1.55	(0.98 – 1.24)	1.45	(1.27 – 1.65)
Rural area	1.10	(1.38 – 1.74)	1.12	(0.99 – 1.28)
**SF-36 Health Status Measures**				
Physical functioning	0.91	(0.89 – 0.94)	1.00	(0.96 – 1.04)
Role-physical	0.96	(0.95 – 0.98)	0.99	(0.97 – 1.01)
Role-emotional	0.98	(0.97 – 0.99)	1.00	(0.98 – 1.02)
Social functioning	0.95	(0.92 – 0.97)	0.99	(0.95 – 1.03)
Bodily pain	0.96	(0.94 – 0.98)	1.03	(1.00 – 1.06)
Vitality	0.91	(0.88 – 0.93)	0.91	(0.88 – 0.95)
Mental health	0.94	(0.92 – 0.97)	1.01	(0.97 – 1.05)
General health	0.94	(0.92 – 0.97)	1.02	(0.98 – 1.06)

## Conclusion

This study is the first population-based study in assessing the consent patterns in a general Asian population. The unique nature of the NHIS allowed us to investigate important characteristics influencing non-consent in a large group of Asian adults who agreed to participate in a national health survey. Our study findings yield important implications for researchers linking survey data to health insurance records in an Asian population. First, people in Taiwan are as likely as people in Western countries to give consent to linkage. The consent rate (88%) observed in a general Taiwanese population, a typical Asian population, is within the range of consent rates observed in Western countries such the USA, the UK, and Australia. Although the consent rate is high for record linkage studies, non-consent still adds data attrition on top of that attributable to non-response of the original survey. Hence, it must be taken into consideration in the initial sample size calculation [2,3,5,6].

Second, contrary to the findings in Western countries, in Taiwan we did not find any discrepancy in gender and self-reported health between individuals who consented and those that refused. Also, whereas ethnic minorities in the UK or the USA were less likely to give consent or there was no difference compared to others, the aborigines in Taiwan showed the opposite. This study illustrates the need to investigate consent patterns in populations with different cultural backgrounds.

Finally, in Taiwan, a typical Asian society, consenters differed significantly from non-consenters in important aspects such as age, education and income level. One plausible explanation is that some of the elderly, illiterate or lower-income participants may be hesitant to give consent due to ineffective communications or lack of trust between them and interviewers. As the non-uniform consent distribution of these variables may be significantly related to utilization and health outcomes, record linkage research restricted to consenters may encounter the risk of miss-characterizing the utilization and health outcomes of the general population and thus lead to selection bias. With other words, having a high consent rate (88%) will not necessarily guarantee full elimination of possible selection bias. As one expects some refusal to participate in a record linkage study of this nature, researchers need to increase sample sizes to account for non-consenters, and they must investigate potential effects of bias using sensitivity analyses. Also, in order to minimize the potential source of bias mentioned in this study, more work is needed to explore strategies for increasing the proportion of participants consenting to linkage of health insurance records in surveys.

## Competing interests

The author(s) declare that they have no competing interests.

## Authors' contributions

NH and YJC were involved in all aspects of this study including conceptualization, analyses, interpretations, and writing of the article. SFS helped to perform the statistical analyses and interpretation of the results. HYC was involved in data collection, analyses and interpretation of the results. All authors read and approved the final manuscript.

## Pre-publication history

The pre-publication history for this paper can be accessed here:


